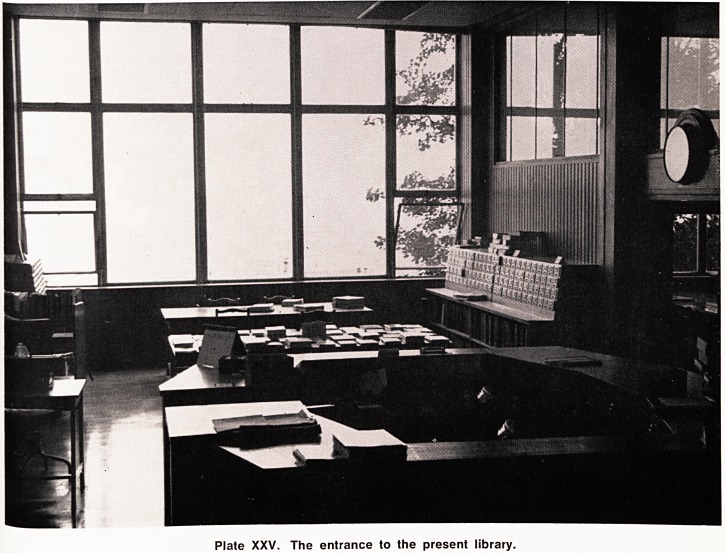# On the History and Growth of the Bristol Medical School Library
*Based on a talk given in the Medical School, June 1970


**Published:** 1970-10

**Authors:** A. E. S. Roberts


### On the History and Growth of the Bristol Medical School Library*

A. E. S. Roberts

After a period of 45 years as a member of the library staff it was felt that I ought to put on record some account of the growth of the library during that time. I would like, if I may, to go further back and say something too about the origins of the library.

But first may I refer to Bristol and its medical connections which, so to speak, set the scene for the establishment of the library. I have two reasons for this, the first is that I am a Bristolian, and the second is that a medical library necessarily forms an integral part of a flourishing medical environment.

For eight centuries or more Bristol has continuously ranked among the leading towns of the country, and in this respect differs from other great provincial centres which exceed it in size and population. Other cities like York and Norwich which equalled it or surpassed it in pre-Reformation days are no longer in the same rank in size and importance, and other cities which now exceed it have attained the front rank only in the last two centuries.

The chief glory of mediaeval medicine lay undoubtedly in the organization of hospitals and nursing. At the end of the 12th century a sudden enthusiasm spread over Europe for building and endowing hospitals and refuges for the sick and infirm, and for two hundred years this fashion continued. The little town of Bristol, like others, was filled with charitable institutions. But what medical men were available?

There were physicians who were taught and licensed by the Universites of Oxford and Cambridge, Montpellier, Paris and the Italian Schools, and there were members of monastic orders who took medical degrees. There were a few high-class surgeons who were examined and licensed by the Italian universities and there were the Barber-Surgeons, who belonged to a guild which was a strange and peculiarly English institution, and which was granted by law the monopoly of surgical practice in the towns and for 400 years educated and licensed almost all the British surgeons. There was such a Barber-Surgeons' Society in Bristol, but the date of its foundation is unknown. We do know, however, that in 1439 the Bristol Guild obtained its licence from the municipal authorities which was required by an Act of 1436. This control went on till 1745 when Parliament separated the London Barbers' Company from the Surgeons and at about the same time separation took place in Bristol. The Bristol Barber-Surgeons' Hall was in Exchange Avenue but it has long been dismantled.

In the early part of the 18th century a second great enthusiasm for founding hospitals suddenly grew up, for the country had been practically without hospitals for two centuries. Although the Act of Elizabeth had authorised hospitals or poor houses for single parishes hardly any had been built till John Cary in Bristol proposed a scheme which combined parishes into a Union and erected a joint hospital supported by voluntary contributions and rate aid. This was confirmed by Act of Parliament in 1696 and St. Peter's Hospital came into being. The extraordinary success of this Corporation of the Poor institution led to the general adoption of the plan throughout the country. (St. Peter's Hospital, among other things, an architectural gem, was completely destroyed by enemy action in 1941). The great voluntary hospitals began with the founding of the Westminster Infirmary in 1719, and some twenty towns soon provided themselves with similar hospitals by local subscriptions. Bristol was about the first town in the English provinces to build and open an Infirmary in 1737. Dispensaries to supply medicines grew up in the same unexpected way all over the country and in this too Bristol shared.

Bristol also was a spa. The reputation of the Hotwell Water goes back to the middle of the 15th century when it was mentioned by William Wyrcestre, and during the 18th century the spa attained the peak of its popularity as a fashionable resort. But towards the close of that century the glories of the Hotwells began to fade and the end was no doubt hastened by the quieter condition of European politics which enabled people to visit the continental spas in safety.

### FAMOUS BRISTOL DOCTORS

A few famous Bristol medical names ought to be mentioned.

Bristol's earliest medical name is John Free, the son of Bristol parents and a noted Greek scholar, who

* Based on a talk given in the Medical School, June 1970

about 1464 was rector of the Church of St. Michael on the Mount (which is adjacent to the medical school building). He studied in Italy where he became a great teacher of medicine at Florence, Padua and Ferrara. The MS copy of his "Cosmographia" in the library of Balliol College, Oxford (where Free took his degree in 1449) was presented by William Wyrcestre, the famous topographer, who spent the last years of his life

**Plate XXI. The Medical Library in 1892 on the opening of the Medical Wing in University Road (now a geography lecture theatre).**

in Bristol practising physic and cultivating medicinal herbs in his house and garden near St. Phillip's Churchyard, where he died about 1484.

Edward Tyson, the great anatomist, was born in Bristol in 1650 and was one of the founders of St. Peter's Hospital.

Thomas Dover (1660-1742), the pupil and friend of Sydenham, and the inventor of "Dover's Powder", was the first physician appointed by the Bristol Corporation of the Poor to take charge of the patients in the newly-opened St. Peter's Hospital in 1696.

Thomas Beddoes (1760-1808), practised medicine in Bristol and embarked upon experimental work with gas inhalation. In 1799 he founded the Pneumatic Institution in Dowry Square and appointed Humphry Davy as Director who anticipated by fifty years the use of nitrous oxide as an anaesthetic in surgery.

Edward Long Fox (1761-1835), was one of the earliest reformers of the treatment of the insane and built a private asylum at Brislington in the early part of the 19th century.

William Budd (1811-1880), Physician to the Royal Infirmary from 1847 to 1862, announced in the 'Lancet' in 1856 his conviction that typhoid fever was spread by contagion and established the fact that infection came from the excreta of the patients. He also, in 1867, put forward the then novel theory that pulmonary tuberculosis was not a constitutional disorder but an infectious disease.

Elizabeth Blackwell (1821-1910) was born in Bristol and was the first woman admitted to the Medical Register.

Robert Fletcher (1823-1912), too, was born in Bristol and entered the Medical School in 1839. He had a lifelong connection with the Index-Catalogue of the Surgeon-General's Library and with the Index Medicus. In 1912 he received the honorary degree of M.D. from the University and his death the same year deprived the world of medicine of its Bibliographer-in-Chief.

There is in the library a book called "Medical World gallery of contemporaries in the field of medical science" published in Berlin about 1925. It consists of sixty portraits of medical men of the 19th century, including such names as Ehrlich, Pasteur and Virchow. There are six Bristol men among those sixty. The first anniversary meeting of the Provincial Medical and Surgical Association (now the B.M.A.) took place in Bristol in 1833 and of the 400 members of the Association at that time 50 were Bristolians.

The success of the Medical School really dates from the foundation of University College (1876) and the affiliation of the School to it, a step which was of some importance in the eyes of the public for with the generous help of the medical profession and support of the public it became possible to erect a wing of the College which was appropriated entirely by the School. There were signs that the people of Bristol were beginning to shake off that lethargy in the matter of science, literature and the arts which had been so conspicuous during the previous half century, and to feel some care for things besides trade, commerce and politics, and the University College and Medical School must be allowed to have had a considerable share in this change.

In the middle of the 19th century Bristol was a city of strongly-marked political opinions. The Hospital

**Plate XXII. Library in old Medical Wing, 1911, after extra shelving had been added.**

(founded in 1832) had from the first been supported by Liberals, the Infirmary by Tories: and soon after permission had been given to the Infirmary to add the prefix 'Royal' to its name (1850) a Hospital supporter made the remark that "The patients who want a sovereign remedy will now go to the Royal Infirmary: but those who want a radical cure will go to the Hospital".

### ORIGINS OF THE LIBRARY

I have called this talk "On the history and growth of the Medical School Library", but the difficulty is where to begin? The Medical School came into being in 1833, but it possessed no library to speak of till 1893 when, on July 1st, the Bristol Medico-Chirurgical Society handed over its books and periodicals to form a joint library, and to this were added the libraries of the Royal Infirmary and of the Hospital, and they called it the Bristol Medical Library: its affairs were controlled by a committee of three members from the Society and three from University College. And yet if the age of the Library means the age of the separate collections of which it is made up, then the Library is well over 200 years old. But the Medical Library, as we know it to-day, dates from this time, i.e., 1893.

The opening of the new wing of University College assigned to the Medical School took place on the 16th November, 1892, and in his Presidential Address to the Bristol Medico-Chirurgical Society on the 11th October, 1893, Greig Smith said: "The Medical School, the natural centre of our profession in this as in every city which possesses a school, provides an admirable home for the Library where we are now met, and will at the same time make substantial additions in funds and in books. The valuable and extensive libraries of the Infirmary and Hospital will be placed on our shelves and we may look forward to a valuable gift from a neighbouring institution (the Museum and Library). Associations and private individuals have generously added to our stock. With some 10,000 volumes either on our shelves or very soon to be there, and some 90 current periodicals on the tables, we may fairly claim to have made a good start towards having a Library worthy of our centre and of ourselves. But the work is little more than started".

Mr. F. Bligh Bond, the architect of this new Medical School in University Road, said of the Library — "The Library is entered under a richly moulded arch, with carved spandrels emblematical of the mysterious principles of life and the healing attributes of the Deity. The room, which is planned to take the associated libraries of the Medical School and the Bristol Medico-Chirurgical Society, is a very handsome one, 50 feet x 30 feet, lit on three sides by mullioned and traceried windows, and dignified by an open timber roof, with panelled compartments, having carved bosses at their intersections, and supported by massive moulded hammer beams, with carved braces and fretted spandrels. These beams rest upon six moulded freestone corbels, bearing shields, on which are cut monograms or initials of some of those who have had a distinguished connection with the School (Dr. Henry Riley, Mr. Henry Clark, Mr. William Herapath, Dr. John Addington Symonds, Dr. Joseph Griffiths Swayne and Dr. William Budd). A handsome freestone chimney piece, of the old baronial type, stands at one end of the room, and the solid ranges of the dark-wood bookshelves complete the circuit of the room. A pleasant effect for reading purposes is produced in this library by the introduction of cathedral glass in all the lights, a pale green tint slightly predominating".

This room is now a geography lecture theatre, and is easily recognisable from this description.

But for all that the Library was now housed in a room magnificent in its proportions and decorations, it was strangely inadequate for growth, although it had been specially designed for the purpose. The walls on three sides of the room were occupied for about three-quarters of their height by windows which, though very beautiful in themselves, considerably lessened the wall-space available for bookshelves and it soon became necessary to erect shelves against the greater part of these windows.

And now let us trace the history of some of these collections which had been combined to form the Medical School Library. This concentration explains how we come to possess such a magnificent collection of the classics of medicine and early printed books, for some of these old libraries were personal collections built up by book-lovers and experts in their professional field.

We now have some 3,000-4,000 items in our rare book collection. At a recent sale of old medical books at Sotheby's very high prices were paid for a large number of them, many of which we have. For instance, the three volumes of Richard Bright's "Reports of medical cases" went for £1,600 and our own copy of this work, being a gift to the library from the author and containing his inscription, is no doubt worth several hundreds more; Carpue's work on plastic surgery of the nose, a slim quarto volume and published no longer ago than 1816, fetched £1,400; Jacques Guillemeau's "The French chirurgerye", 1597, fetched £950; John Mayow's "Tractatus quinque", 1674, fetched £500; and our most precious possession is Vesalius' "De humani corporis fabrica", 1543, which is worth some £6,000. These are only a few examples of the treasures the library possesses.

The library of the Bristol Medico-Chirurgical Society was established in 1890 and was formally opened on the 5th January, 1891, by the President of the Society, Mr. Samual Henry Swayne (1820-1900), as a Medical Reading Room and Reference Library in accommodation provided by the Literary and Philosophical Club at 22 Berkeley Square. A start was made with over a thousand volumes (including a large donation from the B.M.A.), and seventy-nine current periodicals, and it quickly grew as the result of gifts from individuals, grants from the Society and review books and exchange journals through the Bristol Medico-Chirurgical Journal. At this time, as I have said, the Medical School had no library to speak of for its staff, and did not provide books either for its teachers or for its students, so the transfer of the Society's library to the Medical School buildings in 1893 was a very welcome one.

The collections belonging to the Royal Infirmary and the Hospital were incorporated in 1894, mainly through the influence and energy of James Greig Smith. The Infirmary Library had been established in 1826 by an initial gift of books from Richard Smith and Richard Lowe, and there is a printed catalogue in the Medical Library of the contents of the Infirmary Library which shows what a magnificent library it was, being particularly rich in 18th century books.

Soon after the incorporation of the Royal Infirmary and Hospital libraries a further collection of books was presented — this time by the City Authorities. This particular library had had a rather chequered history. It was originally called the Bristol Medical Library when it was founded as a subscription library by Edward Kentish and others in 1832. At the fifth annual meeting in 1836 it was reported that papers had been read at evening meetings, so that it is evident that the members were not confining themselves to library work, and this explains the title of Bristol Medical Library Society, by which the institution was then known. It was housed in a building in Orchard Street, leased from the Corporation at a yearly rental of two pounds. The Library remained there till 1856 when it was transferred to the custody of the Bristol Library, another and older subscription library founded in 1772, which had just moved from King Street to the west wing of the Bishop's College which then stood on ground afterwards occupied by the Art Gallery. The Bishop's College ceased to exist in 1861, but the libraries were able to remain there till 1871 when they were moved to the Museum and Library in Queen's Road. The Museum and Library did not receive the public support that was expected and in 1893 it passed to the ownership of the city. Soon afterwards the City Authorities handed over to the Medical School the medical portion of this library which, of course, contained many of the books from the old Orchard Street collection. I have come across several of these books with the Bristol Medical Library Society label which states that "This book may be kept 10 days" and that "the fine for exceeding this time is 2d per diem".

The Medical Library was now well established, with a Clerk in charge, and here in the Medical School in University Road it remained for twenty years. For most of this time the Library had been a Reference Llibrary only, but in 1908 the University College authorities and the Society accepted the recommendations of a report on the conditions of the Library that members should be allowed to borrow books.

In 1911, when the Library stock had grown to some 22,000 volumes and 250 current periodicals, the University required the Library room for a Council Chamber and in the summer of that year the Library moved to accommodation that had been provided in the east wing of the Blind Asylum. But here the Library's stay was short, for in 1915 the building had to be pulled down to make way for a new University Library and Administration block (now known as Wills Memorial Building), and it was necessary once more to find other quarters for the Medical Library. This was arranged in the old Drill Hall, the entrance to which was from University Road (the Molecular Biology Laboratory now occupies this space). It was not thought the Medical Library would remain here for long, but in fact the library part of the new building was not ready for occupancy till 1923 in which year the medical books were tranferred to their new home (now the Law Library) where it remained for forty years.

**ADMINISTRATION**

From 1912 to 1922 the Medical Library staff, although appointed by the University, was under the orders of the Honorary Medical Librarian of the Bristol Medico-Chirurgical Society, Dr. C. King Rudge (who held the post from 1902 to 1926). The library was managed by a committee of ten members, five appointed by the University and five by the Society. On 21st June, 1922, the Society, at a special meeting, unanimously passed a resolution to make an absolute gift of its library to the University, subject to conditions which would ensure members of the Society, present and future, enjoying full privileges of the Library. A new agreement was completed on the 9th December, 1925, between the Society and the University which provided, among other things, "That the Medical Library shall be under the

**Plate XXIII. The library in the Wills Memorial Building, 1953 (now the Law Library).**

ultimate control of the Librarian of the University who shall be responsible to the Library Committee of the University: that the general management of the Medical Library shall be vested in the University Council who may delegate to the Library Committee power to make and alter regulations for using the same: there shall be a special sub-committee of the Library Committee to be known as the Medical Library Sub-Committee : that the constitution of this sub-committee shall be the Chairman of the Library Committee, the Librarian, the Dean and one other representative of the Faculty of Medicine, two representatives of the Society and the Honorary Medical Librarian who shall be appointed by the Society. The office of Honorary Medical Librarian shall carry no administrative authority, but shall be of an advisory character only".

I hope you will not mind if in places this account now becomes autobiographical for we have now arrived at the time when I joined the Library.

It was on the 19th March, 1925, that I clambered through the hoarding that was still around what is now called the Wills Memorial building and made my way to the Medical Library to start my first day as a library assistant. The building was officially opened by King George V and Queen Mary in June, 1925. I would like first to give an account of the Library from then till I went into the Army in 1941.

The University Library staff in 1925 comprised the University Librarian, two Assistant Librarians and two juniors, and of these the Medical Library claimed one Assistant Librarian and one junior. At this time all University Library administration was done by the Assistant Librarian in charge of the Medical Library, and he acted as Secretary to the University Library. No commercial activity was allowed to affect the academic calm of the Arts Library, as the Wills Memorial Library was then known. All University Library correspondence and other office work was done in the Medical Library office, and all the books and periodicals that came to the University Library, and this included all those for departmental libraries — arts, science and medicine — were recorded and processed by the Medical Library staff. Financial schedules were kept and at the end of each year the Finance Office checked every item spent during the year. These were the days before the introduction of office machinery (the only machine in the Library was a typewriter, and all book-keeping in the Finance Office was done by hand): even the catalogue of the University Library was in manuscript.

I was placed in charge of the Medical Library in 1930 on the death of my immediate superior.

Of course, the Medical School was much smaller then, the annual intake being about 40 students and there were five professors and about 80 teachers, as compared with 26 professors and about 500 teachers now.

At this time the Library regulations allowed medical students the privilege of using the Library for reading and reference only, staff and members of the Bristol Medico-Chirurgical Society were the only people allowed to borrow. The Library was reckoned to be well used if about 20 students were present at one time. There was no Veterinary School yet and there was no separate Dental School building till 1940, dental students having been accommodated at the Infirmary and Hospital for their practical work : their books were in the Medical Library.

The annual grant for the Medical Library in 1926 was £120, of which £10 was spent on books and the rest on periodicals and binding, but fortunately there was £150 in the Michell Clarke Fund. There were in addition departmental libraries in Anatomy, Bacteriology, Pathology, Physiology and Preventive Medicine, each of which had its small library grant. Thirty-five books in 1933 were bought for £32, now the cost of the same number of books would be near £200. To-day we spend between £2,000-£3,000 a year on books alone for the Medical Library.

During the period up to 1941 the World List of Scientific Periodicals had been compiled which gave the location and holdings of periodicals in libraries of the British Isles, and this was used by libraries to borrow from each other under the inter-library lending scheme which had been started by the National Central Library. One of the original rules of this scheme, by the way, was that all such loans were to be read in the borrowing library and not used for home reading. It was chiefly owing to the World List and the inter-library lending scheme that the excellent coverage of medical periodicals in the Bristol Medical School Library became known, and consequently we were asked to lend more and more volumes to other libraries. We prided ourselves on the fact that we always lent more than we borrowed. We still do.

I find it difficult to recapture how the Medical Library fared up to 1941, for my time was almost fully occupied with general University Library administration. It just jogged along, it seems, with no great advance in acquisitions or number of readers. The average annual addition to the stock was about 400 volumes, to-day the number is about 2,000. During the first part of this period the chief concentration was on building up the Main Library of the University.

Just before I left to go into the Army the University Librarian, the late Mr. W. L. Cooper, asked me to show him round my department and indicate the work I was responsible for. He was appalled (!), and in one of his early letters to me while I was away he said that after the War I could choose to be either Medical Librarian or Library Secretary and, of course, I chose Medical Librarian. The secretarial part of my duties was to be done by other people; the only non-Medical Library task I retained after the War was library finance which I continued to do until I could safely hand over to another senior member of the University Library staff.

**POST-WAR PERIOD**

In the post-war period the Medical Library took on a new lease of life. More space became available by the use of part of the extension to the University Library which, though completed in 1939, was not utilised by the University till after the war: it had meanwhile been lent to the Bristol Aeroplane Company. A new, and younger, University Librarian was appointed in 1946. I re-classified all the books in the Medical Library according to the Cunningham scheme. I joined the newly-formed Medical Section of the Library Association and at meetings met other Medical Librarians from all parts of the British Isles. The Library had become well known, as, for a short time during the first year of the War part of the Middlesex Hospital Medical School had been evacuated to Bristol and the Army Blood Transfusion Service was centred upon Southmead Hospital. Undergraduates and postgraduates flooded into the Medical School after the War which had so interrupted medical careers.

At this period the Library contained about 30,000 volumes and 370 current periodicals. The figures now are near 60,000 volumes and 620 journals.

In 1947 a Veterinary School was added to the Faculty of Medicine which involved the Medical Librarian in a great deal of work in the building up of a veterinary library, which now contains some 4,000 volumes and nearly 100 current periodicals. It also meant an increase in the number of students and staff.

In 1950 a large collection of old medical books was added. This came from the Royal United Hospital, Bath, and consisted of an accumulation of medical libraries that had been gathered together by Bath medical men, mainly by Caleb Hillier Parry and from

John Smith Soden, but it had not been added to for many years. It was housed in a damp basement at the Bath Hospital and though some of the volumes were in a very poor condition, the collection was rich in rarities and old classics. The Parry part has a separate manuscript sheaf catalogue and these books are kept together as the "Parry Collection" while the others were incorporated into our already extensive collection of historical works. The University spent over £2,000 on the repair of the old books and I was fortunate enough to get a further £1,800 from the Wellcome Trust to more or less complete this restoration work.

Many years ago I took part in a symposium on "Medical Libraries in 1984" and one or two of my prognostications were very wide of the mark. I said that medical students were divided into two main classes, prospective G.Ps. and prospective consultants, and that after qualification the latter would use the Library a great deal to obtain their higher degrees and the former would be too busy in their practices ever to come near the Library. While this may be true of medical students, the implication that only medical people would be the Library's clients was very wrong, for the Library is now used extensively by biochemists, microbiologists, zoologists, psychologists and others. I also said that the number of medical students would decrease (based on the misleading Willink Report) whereas the annual intake of medical students has doubled over the past few years to 120 and dental students, too, have doubled to 50.

The need for a new Medical School had long been felt and plans for a new building were drawn up and considered in 1958. Stage I of this building included the Medical Library and the move took place in August, 1963. This move to a new home was a welcome one since shelf accommodation had become exhausted in the old library: after all it had been housed there for forty years. Improvements on the old consist of the provision of an ante-library, where none existed before, a better display of the periodicals, adequate glazed cases for the extensive historical collection, light stack-room accommodation with carrels for postgraduates, and increased seating accommodation.

### SERVICES PROVIDED

I would like to give some details of services to readers provided by the Medical Library, for "service" I regard as one of the most important words in a medical librarian's vocabulary.

The first library service given is a very pleasant place to work in. But even at this early stage (the present Library was opened less than seven years ago) we are beginning to experience a shortage of shelf-space and seating accommodation. This is caused partly by the unexpected explosive increase in the annual intake of students and the consequent need for multiple copies of textbooks, and partly by the increased rate of growth of the Library. In the immediate future this shortage is being met by the provision of 24 more chairs and three additional tables in the Senior Library, and arrangements have been made to have the glass fronts removed from the rare book shelves and the collection transferred to the strong-room, thus making available in the reading rooms several hundred additional feet of shelving. In the long term, thought will have to be given to an expansion of the library.

I can remember the time when the Library regulations allowed medical students of the University the privilege of using the Library for reading and reference only, now students of all faculties are allowed to borrow (subject to the control of the Medical Librarian): when the annual intake of medical students was about 40, now it is over 200 including veterinaries and dentals: when all the biochemistry needed by medical students was to be found in Starling, now the very large biochemistry school is headed by two professors: when the Library was reckoned to be well used if about 20 students were present at one time, now it is not uncommon for upwards of 600 personnel to use the Library in one day (in the proportion of 5 undergraduates to 1 postgraduate): we even topped 1,000 in a day on one occasion: and when the Library was administered by a staff of two, now there are six.

Inter-library loans twenty years ago numbered 200 borrowed and 390 lent in a session. The current figures show 1,200 borrowed and 1,700 lent. Xerox, of course, is used whenever appropriate for periodical

**Plate XXIV. The present library in the new Medical School.**

articles to save wear and tear of the volume going through the post and also to save the actual volume going out of the Library. Readers are not charged postage on any inter-library loans we may get for them.

As an indication of the growth of the use of the library, borrowings twenty years ago numbered 2,760 in a session: now they number 26,000. Figures at the end of session 1968-69 showed an increase over the previous session of 60 per cent in the number of readers using the Library and 35 per cent in the number of loans. The extra work entailed was done with no increase in staff. There's "productivity" for you!

A much wider reading habit is evident to-day owing to changes in the curriculum and teaching practices. Also very much more biochemistry is taught these days, and here all biochemistry is taught in the Medical School, so that we have to cope with the needs of science students as well as our own Medical Faculty students. Psychology and Social Science students also attend lectures given by the medical staff, and they, too, make use of the Medical Library.

Medical Library services are offered beyond the confines of Bristol by giving help to hospital libraries in the South West in the matter of providing and checking references and by inter-library loans. Several hospital libraries have been formed or enlarged in the South West, mostly in connection with Postgraduate Medical Centres, and the Bristol Medical School Library acts unofficially as the Regional Central Library. This close and willing co-operation applies to centres at Plymouth, Truro, Exeter, Taunton and Bath, as well as to the hospital libraries within Bristol itself. May I remind you that Greig Smith said in 1893 that a Medical School is the natural centre of the medical profession in a given area, and this is true, for I believe that consultants and other medical people are entitled to a reasonable library service wherever they may work and they should not be penalised just because their work lies in districts far from a good medical library. They treat patients in exactly the same way as those who work in the large medical centres. There is a regular van service provided by some local hospitals for the collection and return of library items on behalf of the hospital staffs, all such borrowings being channelled through the Hospital Librarian or Postgraduate Medical Secretary, who are responsible for such loans. This saves a great deal of the time

**Plate XXV. The entrance to the present library.**

of hospital staffs, particularly those who work on the perimeter of the city, but I am not sure if this is a good thing when some of them hardly ever visit the Library now that their material is delivered to them.

Registrars in the teaching hospitals are regarded 'ipso facto' as tutors in the Faculty of Medicine, so that through membership of the Bristol Medico-Chirurgical Society (which includes many general practitioners), through the Hospital Libraries and Postgraduate Medical Centres, the facilities of the Medical Library are available to practically all medical people in the South-West.

After working for 45 years in the Medical Library it is time I retired and while I shall be glad to do so it is nevertheless very gratifying to me to be told by many people that I shall be missed. My successor (Mr. Brian Jones from Sheffield University) has been appointed and is to take up his duties on August 1st, 1970. I do not know what, if any, changes he will make but I hope he will agree with me that in a library 'service' is of paramount importance, restrictions should be kept to a minimum (subject to the convenience of the many), and that a librarian should not regard the library as his own — it belongs to the users — he is only the custodian.

I hope I have covered what seems to me to be three phases in the history of the Library, up to 1922 when it was indeed the Medical School Library; for a period from 1923 when it lost its identity somewhat and seemed to be just the medical section of the University Library, and from 1963 to date when, though administratively part of the University Library System, it enjoys once again in its new home a more autonomous existence as the Medical School Library.

**References**

Prichard, A. (1892). Early history of the Bristol Medical School. Bristol Medico-Chirurgical Journal, **10,** 264-291.

Mitchell, R. J. (1955). John Free. Longmans, London.

Parker, G. (1922), Early Bristol medical institutions. Transactions of the Bristol and Gloucestershire Archaeological Society, **44,** 155-178.

Parker, G. (1933). Schola Medicinae Bristol. Wright, Bristol.

Smith, G. M. (1917). A history of the Bristol Royal Infirmary. Arrowsmith, Bristol.

Griffiths, L. M. (1911). The Bristol Medical Library. Bristol Medico-Chirurgical Journal, **29,** 335-344.

Rudge, C. K. (1912). The Bristol Medical Library, Bristol Medico-Chirurgical Journal. **30,** 344-350.

Smith, J. G. (1893). Modern medical journalism. Bristol Medico-Chirurgical Journal. **11,** 218.

## Figures and Tables

**Plate XXI. f1:**
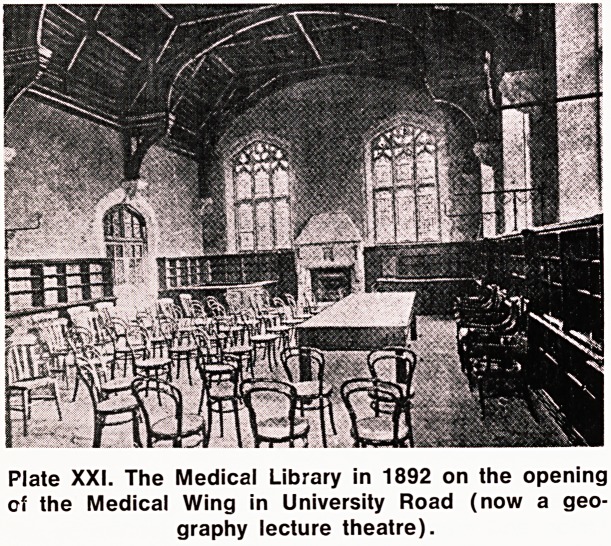


**Plate XXII. f2:**
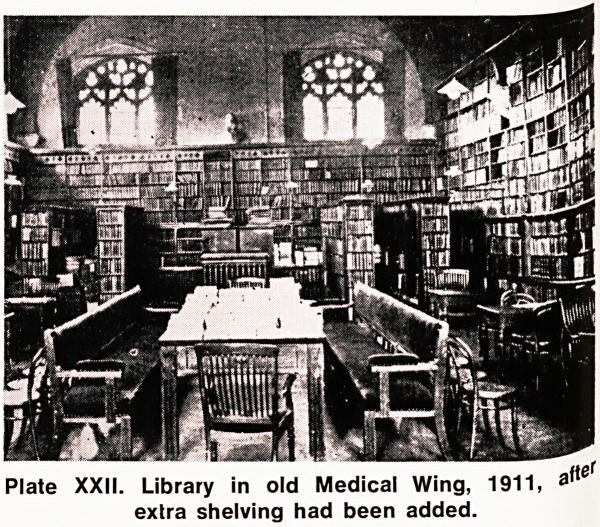


**Plate XXIII. f3:**
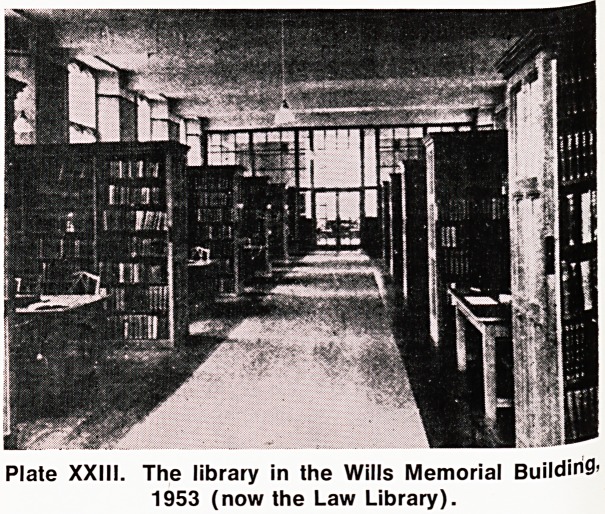


**Plate XXIV. f4:**
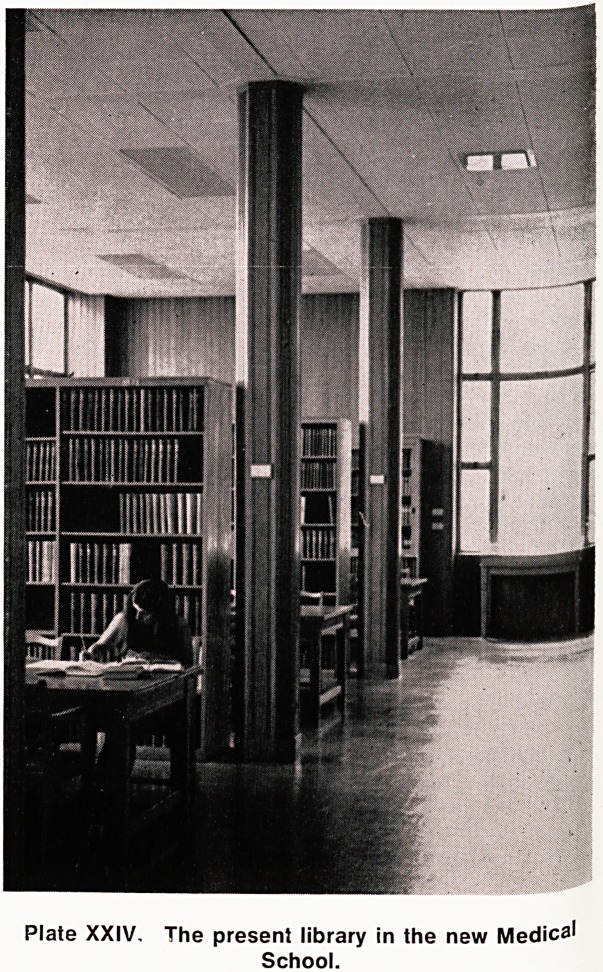


**Plate XXV. f5:**